# Cells from the hematopoietic lineage are only present transiently during healing in a mouse model of non-severe burn injury

**DOI:** 10.1186/s13287-015-0130-1

**Published:** 2015-07-24

**Authors:** Suzanne Rea, Andrew Stevenson, Natalie L. Giles, Fiona M. Wood, Mark W. Fear

**Affiliations:** Burn Injury Research Unit, School of Surgery, University of Western Australia, 35 Stirling Highway, Crawley, WA 6009 Australia; Fiona Wood Foundation, Fiona Stanley Hospital MNH (B) Main Hospital CD15, Level 4, Burns Unit 102-118 Murdoch Drive, Perth, Murdoch 6150 WA Australia; Burns Service of Western Australia, WA Department of Health, Fiona Stanley Hospital MNH (B) Main Hospital CD15, Level 4, Burns Unit 102-118 Murdoch Drive, Perth, Murdoch 6150 WA Australia

## Abstract

**Introduction:**

The aim of our study is to identify the contribution of hematopoietic-derived cells to burn-wound healing in a non-severe injury. There are many conflicting reports of the contribution of bone marrow-derived cells to wound healing and whether these are hematopoietic or mesenchymal in origin. The role of hematopoietic lineage cells is investigated in this study in the context of the response to burn injury.

**Methods:**

Transgenic mice expressing the LacZ reporter gene in all cells of the hematopoietic lineage underwent a non-severe full-thickness burn injury (8 % of total body surface area). Wounds were assessed for LacZ-positive cells at days 7, 14, and 28 post-injury by using whole-mount staining. Cells were also cultured from the wounds at each time point and analysed for expression of fibroblast and myofibroblast markers.

**Results:**

At day 7, positive cells were identified in the wounds representing the inflammatory response. Some dermal cells were also identified at this early stage. At day 14, positive cells were also identified and were cultured from the wound tissue samples. However, by day 28, no positive cells could be detected or cultured from the healed wound tissue. Isolated LacZ-positive cells did not express collagen 1 or α-smooth muscle actin proteins, indicating that they had not differentiated into dermal fibroblast-type cells.

**Conclusions:**

In this model of burn injury, hematopoietic lineage cells were present in the healing wound only transiently and did not appear to contribute to the long-term scar population. This is in contrast with reports demonstrating that fibrocytes contribute a long-term sustained population in scar tissue. This work demonstrates that in a non-severe burn injury model there is a sustained transient contribution of hematopoietic cells to the healed wound. Further characterisation of the types and extent of wounding required to establish a long-term hematopoietic response will be important in determining future cell-based therapies.

## Introduction

Many reports support an important role of bone marrow-derived cells, including fibrocytes, in the post-inflammatory phase of wound repair [1]. Fibrocytes have been implicated in the healing process in diverse tissues, including liver, lung, and skin, and an array of roles have been attributed to these cells in long-term repaired tissue such as hypertrophic scar [2]. Fibrocytes are thought to be cells of hematopoietic origin, differentiating into mesenchymal cell types as they migrate into sites of injury [3]. However, others have shown no long-term contribution of cells of bone marrow origin in healed wounds [4, 5]. Therefore, whilst the evidence supporting a key transient role in wound healing for fibrocytes is substantial, the issue of whether cells of hematopoietic lineage have a long-term role in healed tissue is not clear [6]. Here, we have used the Vav-Cre transgenic mouse model to monitor the fate of cells of hematopoietic lineage after moderate burn injury. C57BL/6 J Vav-Cre transgenic mice, which express Cre recombinase under the control of the Vav promoter, restricting expression to all hematopoietic lineage cells and endothelial lineages [7, 8], were crossed with the ROSA26R-LacZ reporter strain [9]. Offspring were genotyped, and recombinant mice expressing LacZ restricted to the hematopoietic lineage received a 1.9-cm diameter full-thickness burn injury (as previously described [10]).

## Methods

### Ethical approval

All animal experiments were carried out with approval by the relevant institutional animal ethics committees (Murdoch University, Perth, Australia, approval #R2080/07; Royal Perth Hospital animal ethics committee approval #R13/07), and all experiments were conducted in accordance with the National Health and Medical Research Council’s Australian Code of Practice for the Care and Use of Animals for Scientific Purposes.

### Genotyping

For parental genotyping, tail-tip genomic DNA was prepared [11]. Cre-positive animals were genotyped by using polymerase chain reaction primers and conditions as previously described [7]. ROSA26LacZ animals were genotyped by using primers and conditions previously described [9].

Recombinant Vav-CreRosa26LAcZ offspring to be used for burn injury experiments underwent a pre-injury full-thickness 3-mm punch biopsy. This biopsy was taken at the site of the burn injury and immediately prior to the injury. Biopsies underwent whole-mount staining for LacZ (described below) and were sectioned and analysed. Those animals with detectable LacZ in circulating and endothelial cells only were assessed post-injury. Other animals either with no detectable expression or with expression not restricted to the hematopoietic and endothelial lineage (chimeric mice) were euthanased prior to analysis [8].

### Mouse burn injury and sample preparation

The murine burn wound model is a contact thermal full-thickness injury on the dorsum of the mice and is approximately 8 % of total body surface area [10]. At days 7, 14, 21, and 28 post-injury, control animal groups were euthanased (n = 5 per group per time point). The entire wound, including adjacent 3-mm skin margins, was excised. The wound was bisected along the cranial caudal axis. One half of the sample was processed for whole-mount LacZ staining, and the second half of the sample was prepared for cell isolation and culture.

### LacZ whole-mount staining and histology

Tissue was washed three times for 20 min at room temperature and incubated overnight at 37 °C in the dark in LacZ staining buffer: 2 mM MgCl_2_, 5 mM K_3_Fe(CN)_6_, 5 mM K_4_Fe(CN)_6_, 5-bromo-4-chloro-3-indolyl-β-D-galactopyranoside (X-gal) 1 mg/ml in phosphate-buffered saline (PBS). Samples were then washed three times in 3 % dimethyl sulfoxide/PBS for 10 min, fixed in 4 % paraformaldehyde, and paraffin-embedded. Tissue sections (5 μm) were deparaffinised, stained in Eosin stain for 45 sec, and imaged under a light microscope. For counting LacZ-positive dermal cells at day 14, three areas of 0.1 mm^2^ in each section were assessed for positive and negative dermal cells (excluding hair follicles and endothelial cells). Two sections were counted for each wound for a total of five animals (10 sections, three areas per section).

### Cell isolation and characterisation

Tissue was dispersed into a single cell suspension as previously described [10]. The single cell suspension was then washed thoroughly before being plated in T25-cm^2^ tissue culture flasks in Dulbecco’s minimum essential medium/F12 Glutamax supplemented with 10 % fetal bovine serum, 0.5 μg/ml Fungizone antimycotic liquid, 100 μg/ml kanamycin, 100 U/ml penicillin, and 100 μg/ml streptomycin (Invitrogen, Carlsbad, CA, USA) and incubated at 37 °C in 5 % CO_2_. After 24 h, the cells were washed with PBS (pH7.4), and fresh media minus Fungizone and kanamycin was added to the cells. Once the flask was confluent, cells were seeded onto round coverslips fitted into six-well plates before being fixed in glutaraldehyde (diluted 1:100 PBS pH 7.4) and stained by using a LacZ staining solution (2 mM MgCl_2_, 5 mM K_3_Fe(CN)_6_, 5 mM K_4_Fe(CN)_6_, X-gal 1 mg/ml in PBS) overnight at 37 °C in a dark chamber.

### Characterisation of LacZ-positive cultured cells

Isolated cells underwent further characterisation by staining for fibroblast markers in addition to LacZ. Immunofluorescence was conducted by using a rabbit anti-collagen I antibody (Abcam, Cambridge, MA, USA) to stain for collagen type 1-producing fibroblasts and rabbit anti-α-smooth muscle actin antibody (Abcam) for myofibroblasts. Secondary antibody goat anti-rabbit IgG Alexa Fluor 488 (Molecular Probes, Eugene, OR, USA) was used. Slides were viewed by using a fluorescence microscope at the Centre for Microscopy, Characterization and Analysis at the University of Western Australia.

## Results

**Fig. 1:**
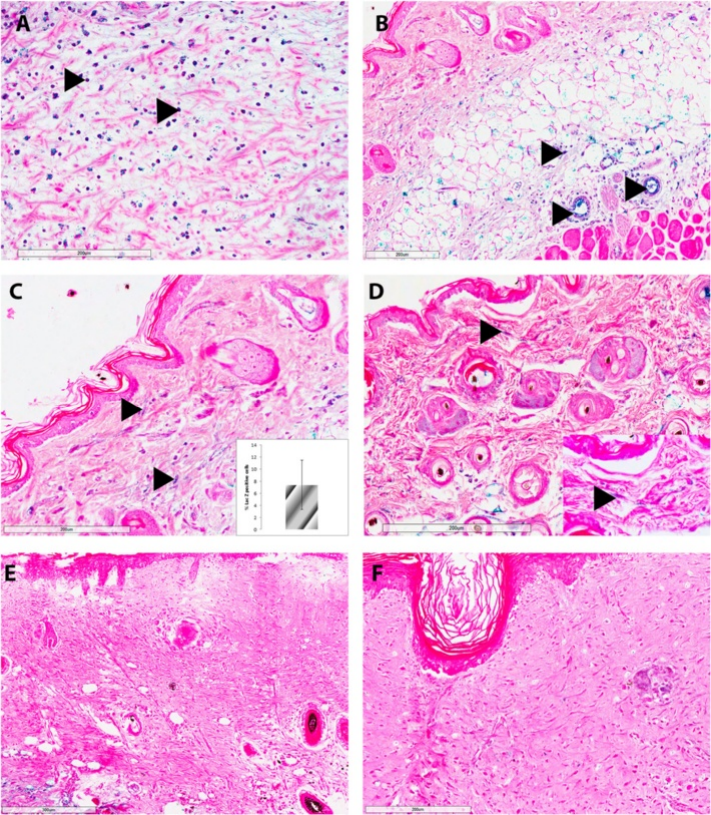
LacZ-positive cells are detected in the wound up to day 14 after moderate burn injury. LacZ-positive cells are present in the wound at day 7 (**a**, **b**) and day 14 (**c**, **d**). At day 7, substantial inflammatory infiltrate in the skin and loose connective tissue below the panniculus carnosus (**a**, **b**) are observed. By day 14, positive cells remain and some cells in the dermal layer are positive for LacZ. (**c**) *Inset* shows percentage of positive LacZ dermal cells at day 14 ± standard deviation. **d** *Inset* shows higher magnification of LacZ-positive dermal cells. At day 21 (**e**) and day 28 (**f**), LacZ-positive cells other than endothelial or immune cells are not detected. At day 14, 14 % ± 4.1 % of interfollicular dermal cells appear to be positive for LacZ (**c**). *Arrows* indicate LacZ-positive cells

Whole wound tissue was isolated and stained for the expression of LacZ post-injury. At day 7 post-injury, there is a substantial inflammatory infiltrate both in the dermal layer and in the loose connective tissue below the panniculus carnosus (Fig. 1a, b). At day 14 post-injury, there is positive staining for LacZ in cells within the dermal compartment (Fig. 1c, d). These cells appear in the circulation and as inflammatory cells, but cells can also be observed in the dermis and they appear to have the distinct fibroblast spindle shape (Fig. 1d, inset). There is a high degree of variation within the wound as to the percentage of LacZ-positive cells are in the dermis with an average value of 7.4 % ± 4.1 % of cells in the dermis being LacZ-positive at day 14 (mean ± standard deviation, Fig. 1c inset). At days 21 and 28 post-injury, no LacZ-positive cells were detected in the interfollicular skin (Fig. 1e, f, respectively). Positive expression at days 21 and 28 consisted solely of hematopoietic cells and blood vessels consistent with the normal expression of Vav-Cre recombinant mice.

**Fig. 2:**
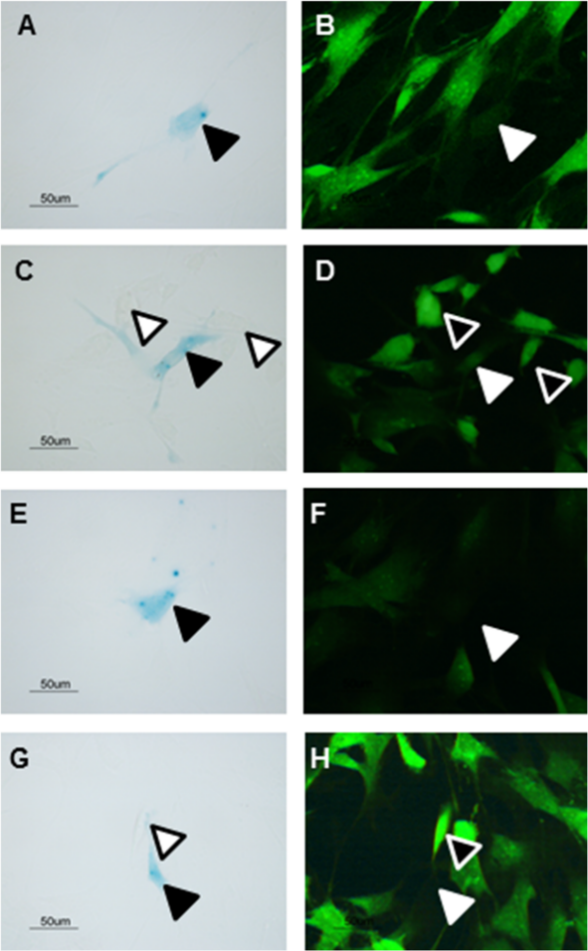
Isolated LacZ-positive cells do not express collagen I or α-SMA. Cells isolated at day 14 were stained for LacZ and collagen I (**a**-**d**) or α-SMA (**e**-**h**). Positive cells identified for LacZ expression (**a**, **c**) do not stain for collagen I expression (**b**, **d**). Other isolated cells not positive for LacZ expression are collagen I-positive (**b**, **d**). Positive cells identified for LacZ expression (**e**, **g**) do not stain for α-SMA expression (**f**, **h**). Other isolated cells not positive for LacZ expression are α-SMA-positive (**f**, **h**). *α-SMA* alpha-smooth muscle actin

Tissue samples were also used to isolate dermal cells. Interestingly, whilst LacZ-positive cells were isolated from day 14 tissue samples, no lacZ-positive cells were isolated from day 21 or day 28 samples (data not shown). LacZ-positive cells have fibroblast-like morphology (Fig. 1d). However, they do not appear to express either collagen I (Fig. 2a-d) or α-smooth muscle actin (α-SMA) (Fig. 2e-h). This strongly suggests that they are not of a true fibroblast phenotype.

## Discussion

The data clearly show a transient population of hematopoietic cells within the healing wound. This appears to be predominantly during the acute inflammatory phase, and a small number of cells persist longer in the healing dermis. Immunohistochemistry suggests that these cells are not of a normal fibroblast phenotype. Therefore, it is more likely that they represent a residual population of differentiated immune cells, most likely monocytes differentiating into macrophages which are removed at a later point in healing.

From this study, we conclude that there is no long-term contribution to healed skin tissue of hematopoietic lineage cells. The use of this transgenic model allows tracking of cell fate irrespectively of subsequent differentiation. Other reports have suggested that the differentiation of hematopoietic lineage cells, once in the wound, changes the markers expressed (for example, loss of CD45 expression) and this masks their continued presence [12, 13]. However, this study shows clearly that no cells of hematopoietic lineage persist in this model of injury.

This model used an injury of approximately 8 % of total body surface area. In a similar small injury model using parabiotic mice, an early response of circulating cells was identified in the wound, and no long-term contribution was observed [5]. However, the circulating cells were identified as expressing fibroblast or myofibroblast markers, although this appeared to be transient and declining by day 7 post-injury, suggesting that by day 14, similar to our observations, there was unlikely to be expression of these markers.

Both of these studies used small injury models, and it is possible that, in injuries of greater extent, trans-differentiation and long-term persistence of hematopoietic cells occur. However, this would restrict the contribution of hematopoietic cells to only severe injuries, contradicting other reports to date [14]. It is also possible that the murine model does not accurately reflect the human response. It has been shown that there are significant differences in the injury response between these organisms [15]. However, many other studies have implicated fibrocytes in long-term wound repair using a mouse model [1] and this is unlikely to underpin the lack of hematopoietic cells observed in this model when compared with some other studies.

Recently, a biphasic response of fibrocytes to a burn injury was demonstrated in a porcine model of injury [16]. This study demonstrated an acute-phase response followed by the absence of fibrocytes until day 56 post-injury. Owing to the shorter time frame of sampling, the possibility that fibrocytes would have been found in later scar samples cannot be discounted in this study.

In this study, we hypothesise that the resident cells observed in the dermis at day 14 were most likely longer-lived macrophage-type cells. There is evidence for macrophages or monocyte precursors differentiating into fibrocytes and fibroblast cells particularly in the context of wound healing [17, 18]. There is also evidence that the monocyte-macrophage-fibroblast lineage is closer to a continuum rather than distinct lineages of cells [13]. However, these cells do not express fibroblast markers, and so although the morphology suggests that they are similar to fibroblasts, they are unlikely to be functioning in the wound in a similar manner. It is possible that these cells are altered in phenotype during culture and that this masks the true cell phenotype. However, other LacZ-negative cells do express typical fibroblast markers, and fibroblasts are commonly cultured ex vivo without significant phenotypic changes. Therefore, although it is unlikely that the LacZ-positive cells are fibroblastic in origin, changes to their phenotype in culture cannot be ruled out. This is also an important consideration for further characterisation studies. Further characterisation of these cells will be important to better understand any potential role in healing.

## Conclusions

This study demonstrates only a transient population of hematopoietic lineage cells present in healing after non-severe burn injury in this mouse model. The use of this well-characterised transgenic model circumvents many of the limitations of other studies with respect to the influence of cell differentiation, cell characterisation, and phenotype assessment on the interpretation of results. It will be important to use this model to assess other injury extents (e.g., severe burn injury) and modalities to determine whether the response is uniform across injury etiology and severity. In light of the recent study showing a biphasic response, it will also be important to extend the time frame of these experiments to assess for a possible re-influx of hematopoietic-derived cells. Finally, it will be critical to characterise the phenotype of the cells identified and to fully assess the role played in the healing wound. A more complete understanding of the origin and role of different cell types in wound responses will ultimately be critical to the future of successful cell-based therapies in the field.

## Abbreviations

LacZ: Gene encoding beta-galatosidase
PBS: Phosphate-buffered saline
X-Gal: 5-bromo-4-chloro-3-indolyl-β-D-galactopyranoside
